# Multi-locus evaluation of gastrointestinal bacterial communities from *Zalophus californianus* pups in the Gulf of California, México

**DOI:** 10.7717/peerj.13235

**Published:** 2022-07-08

**Authors:** David Ramirez-Delgado, Francesco Cicala, Ricardo A. Gonzalez-Sanchez, Rosalia Avalos-Tellez, Elena Solana-Arellano, Alexei Licea-Navarro

**Affiliations:** 1 Marine Ecology Department, CICESE, Ensenada, Baja California, México; 2 Biomedical Innovation Department, CICESE, Ensenada, Baja California, México; 3 Comisión Nacional de Areas Naturales Protegidas, Secretaría de Medio Ambiente y Recursos Naturales, Bahia de los Angeles, Baja California, México

**Keywords:** Microbiome, Pinniped, Bacterial consortium, *Zalophus californianus*, Gulf of California

## Abstract

**Background:**

The gastrointestinal (GI) bacterial communities of sea lions described to date have occasionally revealed large intraspecific variability, which may originate from several factors including different methodological approaches. Indeed, GI bacterial community surveys commonly rely on the use of a single hypervariable region (HR) of *16S rRNA*, which may result in misleading structural interpretations and limit comparisons among studies. Here, we considered a multi-locus analysis by targeting six HRs of *16S rRNA* with the aims of (i) comprehensively assessing the GI bacterial consortium in rectal samples from *Zalophus californianus* pups and (ii) elucidating structural variations among the tested HRs. In addition, we evaluated which HRs may be most suitable for identifying intrinsic, structurally related microbiome characteristics, such as geographic variations or functional capabilities.

**Methods:**

We employed a Short MUltiple Regions Framework (SMURF) approach using the Ion 16S™ Metagenomic Kit. This kit provides different proprietary primers designed to target six HRs of the *16S rRNA* gene. To date, the only analytical pipeline available for this kit is the Ion Reporter™ Software of Thermo Fisher Scientific. Therefore, we propose an in-house pipeline to use with open-access tools, such as QIIME2 and PICRUSt 2, in downstream bioinformatic analyses.

**Results:**

As hypothesized, distinctive bacterial community profiles were observed for each analyzed HR. A higher number of bacterial taxa were detected with the V3 and V6–V7 regions. Conversely, the V8 and V9 regions were less informative, as we detected a lower number of taxa. The synergistic information of these HRs suggests that the GI microbiota of *Zalophus californianus* pups is predominated by five bacterial phyla: *Proteobacteria* (~50%), *Bacteroidetes* (~20%), *Firmicutes* (~18%), *Fusobacteria* (~7%), and *Epsilonbacteraeota* (~4%). Notably, our results differ at times from previously reported abundance profiles, which may promote re-evaluations of the GI bacterial compositions in sea lions and other pinniped species that have been reported to date. Moreover, consistent geographic differences were observed only with the V3, V4, and V6–V7 regions. In addition, these HRs also presented higher numbers of predicted molecular pathways, although no significant functional changes were apparent. Together, our results suggests that multi-locus analysis should be encouraged in GI microbial surveys, as single-locus approaches may result in misleading structural results that hamper the identification of structurally related microbiome features.

## Introduction

The California sea lion (*Zalophus californianus*) is the predominant pinniped species in in the Gulf of California (GoC). However, a recent survey revealed a notable population decline over nearly three decades from approximately 43,834 (1991) to 15,291 (2019) sea lions (Adame et al., 2020) distributed among thirteen islands of GoC (Szteren, Aurioles & Gerber, 2006; Masper et al., 2019). Due to this rapid population decline, the California sea lion is now listed as Endangered on the Red List of the International Union for Conservation of Nature (IUCN) and by the Official Mexican Standard (NOM-059-SEMARNAT). Although the California sea lion is now endangered, limited information is available regarding the current status of the remaining populations.

Over the last few decades, research on GI bacterial consortia has grown, leading to the conclusion that GI bacteria and their associated gene pools (hereinafter referred to as the GI bacterial microbiome) play pivotal roles in determining host health (Libertucci & Young, 2019). Although multiple studies have assessed GI bacterial compositions in sea lions and other pinnipeds species, much remains to be understood. The GI bacterial communities in pinnipeds commonly appear to be composed of only a few phyla, such as Firmicutes, Bacteroidetes, Fusobacteria, and Proteobacteria (Lavery et al., 2012; Smith et al., 2013; Delport et al., 2016; Numberger et al., 2016; Bai et al., 2021). Moreover, the consistent and frequent detections of these taxa in both pups and adults across studies (Nelson et al., 2013; Smith et al., 2013; Tian et al., 2020) have prompted several authors to propose the existence of a common bacterial “*core*” (Numberger et al., 2016; Pacheco-Sandoval et al., 2019; Acquarone, Salgado-Flores & Sundset, 2020).

Despite the probable existence of a bacterial core in pinniped GI microbiomes, huge intra- and interspecific variations in bacterial taxa abundance have also been reported, the causes of which remain poorly understood although they are likely associated with both specific ecological and biological preferences as well as differences in methodological approaches among studies (Milani et al., 2013; Acquarone, Salgado-Flores & Sundset, 2020). For example, diet-driven variations in bacterial microbiotas have been reported in several pinnipeds species and appear to be the principal factors that influence the overall bacterial composition (Aurioles et al., 1984; Porras-Peters et al., 2008; Maslowski & Mackay, 2011; Delport et al., 2016; Masper et al., 2019; Pacheco-Sandoval et al., 2019). Nevertheless, the possibility that methodological differences may also explain the differences in bacterial abundances among studies remains poorly investigated (Acquarone, Salgado-Flores & Sundset, 2020).

In recent years, advances in sequencing technology have generated new and powerful tools to assess bacterial biodiversity in almost any source of environmental DNA, including the GI tracts of host species. In particular, metabarcoding (*i.e*., the taxonomic characterization of environmental communities by analyzing short DNA sequences from one gene) is considered to be one of the most effective approaches to evaluate the diversity of bacterial communities (Creer et al., 2016). Usually, metabarcoding studies focus on one hypervariable region (HR) of the *16S rRNA* gene enclosed by conserved regions (Sperling et al., 2017; Fuks et al., 2018) that is targeted by universal primers. The selected HRs are analyzed to determine the bacterial consortium present in a given sample (Fuks et al., 2018). However, several limitations reduce the reliability of metabarcoding surveys.

To date, no consensus has been reached regarding which of the nine hypervariable *16S rRNA* regions should be targeted to characterize bacterial communities (Kumar et al., 2011; Guo et al., 2013; Barb et al., 2016); while sequencing the entire 16S gene remains costly and time consuming (Sperling et al., 2017). Furthermore, targeting different HRs limits comparisons between studies and weakens any inferences and conclusions that arise (Barb et al., 2016). The detection of certain bacteria may also be biased due to the use of different primer pairs (Brooks et al., 2015). Any set of primers may exhibit a stronger affinity for one bacterial taxon over another, hampering the detection of other relevant species (Gofton et al., 2015; Sperling et al., 2017). These region-related limitations have led Fuks et al. (2018) to propose the Short MUltiple Regions Framework (SMURF). This approach combines the genetic information of different *16S rRNA* regions with the aim of reducing the likelihood of false negative or false positive outcomes.

In this study, to accurately characterize the GI bacterial communities of California sea lion pups and evaluate the extent to which HR-related variance may explain structural changes in the GI bacterial microbiome, a SMURF approach was employed using the Ion 16S™ kit (Thermo Fisher Scientific, Waltham, MA, USA). This kit includes six proprietary primers targeting the V2, V4, and V8 regions in one multiplex PCR reaction while V3, V6–V7, and V9 regions are targeted in a second reaction. Given that distinctive HR-related bacterial consortia were observed, we also evaluated which of the tested HRs were most likely to be suitable for identifying structurally related microbiome features, such as geographic patterns or functional capabilities. To address these questions, we employed an in-house analytical pipeline to integrate open access bioinformatics platforms, such as QIIME2 (Anderson, 2001) and Picrust2 (Douglas et al., 2019), in downstream analyses.

## Materials and Methods

### Sample collection

**Figure 1:**
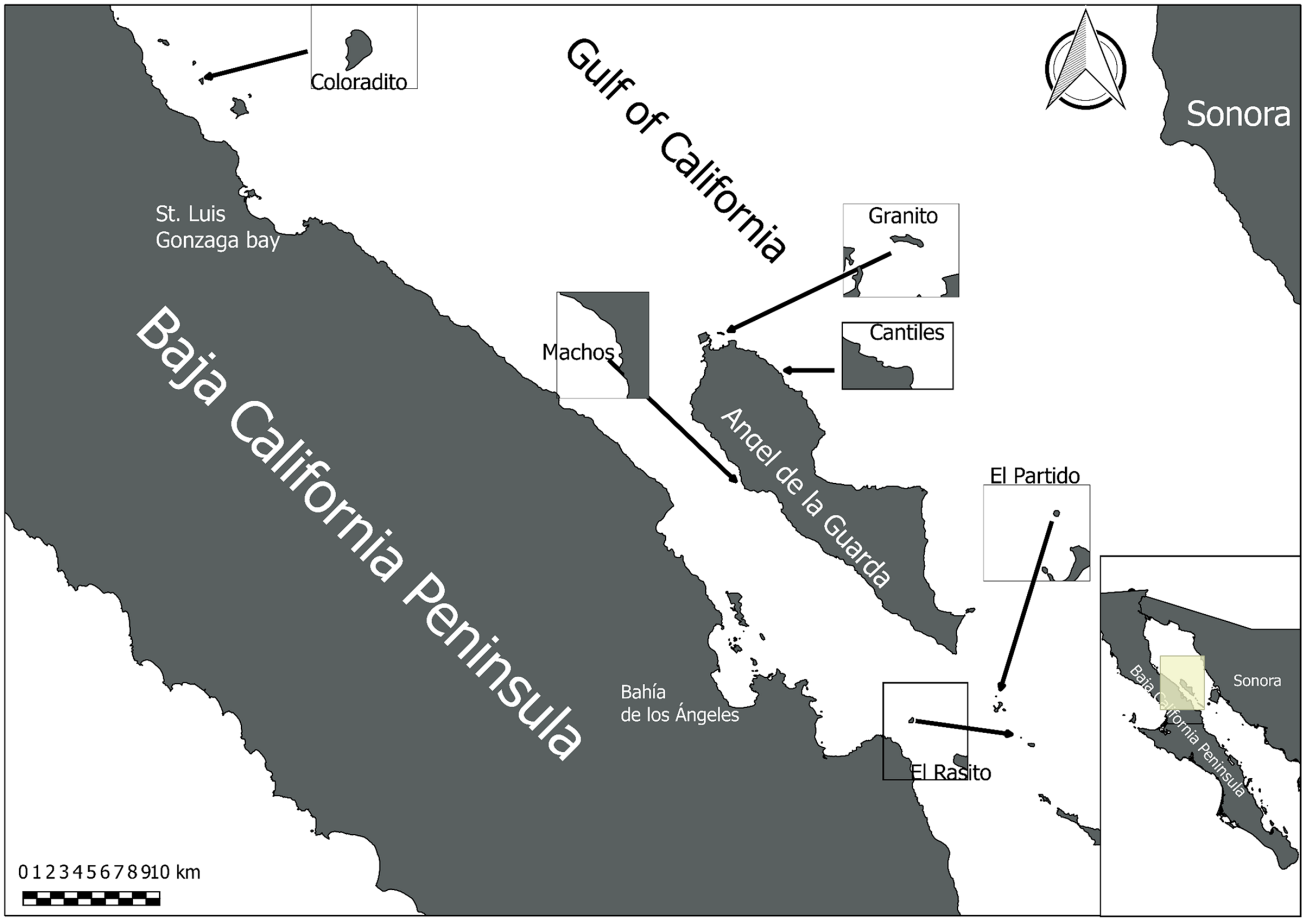
Sampling area. Midriff Islands Region of the Gulf of California in which the six sampled sea lion rookeries (Coloradito, Granito, Cantiles, Machos, Partido, and Rasito) are located.

A total of 54 rectal cotton swabs (RCS) were collected from healthy and randomly selected sea lion pups (2–3 months old) located in six rookeries (*n* = 9 FCS per rookery) in the Gulf of California (Fig. 1). The RCS samples were collected in compliance with Mexican wildlife regulations (SGPVA/DGVS/003083/18). Before sampling, all pups were manually restrained and anesthetized with isoflurane (5%) to reduce mobility and prevent pain. Anesthetic agents were administered by veterinarians of the African Safari Zoo (Puebla, Mexico). All collected RCS were preserved in liquid nitrogen until DNA extraction.

### DNA extraction and purification

Due to the complexity of fecal samples, DNA was extracted and purified using both the Wizard Genomic DNA purification kit (Promega Corporation, Madison, WI, USA) and the PureLink Invitrogen Genomic DNA kit (Thermo Fisher Scientific, Waltham, MA, USA) with protocol modifications. Briefly, 600 μL of nuclei lysis solution was added to 1.5-mL microcentrifuge tubes containing the frozen FCS heads. The tubes were incubated for 25 min at 80 °C. Contaminating RNA was degraded by adding 3 μL of RNAse solution and incubating the tubes for 30 min at 37 °C. Protein was precipitated by adding 600 μL of protein precipitation solution and incubating the tubes on ice for 5 min. Precipitated organic matter was removed by centrifugation for 3 min at 16,000 rpm, and the supernatants were transferred to new, sterile tubes. The DNA was precipitated by adding 600 μL of isopropanol to the supernatants, followed by inversion mixing and centrifugation for 2 min at 16,000 rpm. The supernatants were discarded, and the pellets were washed by adding 600 μL of molecular grade ethanol. The tubes were centrifuged at 16,000 rpm for 2 min before the ethanol was carefully removed and then drained at room temperature for 10–15 min to remove all residual ethanol. The DNA was resuspended in 50 μL of nuclease free water (NFW) and stored overnight at −20 °C. Final DNA purification was achieved following manufacturer protocols with 700 μL of wash buffer 1; wash buffer 2 was used to wash the DNA. A NanoDrop spectrophotometer (Thermo Fisher Scientific, Waltham, MA, USA) was used to measure the DNA concentration and quality before visualization in agarose gel (1.5%).

### Sequencing and reference library construction

Prior to sequencing, the DNA extracts from RCS collected in the same rookery were quantified, normalized, and pooled in equal amounts, resulting in 18 pooled DNA samples. We amplified these samples using an Ion 16S™ Metagenomics kit (Thermo Fisher Scientific, Waltham, MA, USA) following manufacturer protocols. Amplification involved 25 PCR cycles with Ion Xpress Barcoded adapters. Emulsion PCR employed OneTouch™ 2,400-bp chemistry. Sequencing was performed on an Ion Torrent PGM using an Ion 510 chip Kit (Thermo Fisher Scientific, Waltham, MA, USA) with 400-bp read lengths. Torrent Suite v. 4.2.1. Ion Reporter Metagenomics 16S software (v. 5.2) was used for early read trimming and processing according to the following quality criteria: minimum base-calling error of Q20, no barcode mismatches, ≤3 primer mismatches, and length ≥150 bp. Primer detection was set to single end (minimum 90% alignment coverage between hit and query sequences). The raw DNA libraries were deposited in the National Center for Biotechnology Information (NCBI) database (BioProject PRJNA715917).

### Query library preparation and bioinformatic analyses

Raw reads were initially trimmed with Trimmomatic (v. 0.38; Bolger, Lohse & Usadel, 2014) with the following parameters: Leading 3; Trailing 3; Slidingwindow 4:15; and Minlen 150. The trimmed reads were then imported into Quantitative Insights Into Microbial Ecology (QIIME 2; v. 2020.6; Bolyen et al., 2019). Dada2 (Callahan et al., 2016) was employed for denoising and detecting chimeras before the reads were clustered into amplicon sequence variants (ASVs; 100% similarity). The ASVs from the same HRs were filtered by de-novo clustering using VSEARCH (Rognes et al., 2016) and trained on each 16S rDNA reference library in the Ion Reporter Software. The ASVs were aligned with MAFFT (v. 7; Katoh & Standley, 2013), and the phylogenetic reconstruction was carried out with fastTree (v. 2.1; Price, Dehal & Arkin, 2010). The taxonomic position of each ASV was obtained using the BLAST+ consensus taxonomy classifier (Camacho et al., 2009) trained on the SILVA database (v. 132; Quast et al., 2013). To minimize the inclusion of spurious ASVs in the final data sets, singletons and ASVs composed of less than a specific number of HR-library reads (ranging from 15 for V2 to 72 for V8) were removed. Each variable region were then rarefied at the minimum sample read depth using the QIIME 2 core-metrics-phylogenetic plugin, and the resulting abundance tables were used in downstream analyses.

### Ecological and functional prediction analyses

Bacterial communities were initially evaluated *via* rarefaction curves with the median read frequency used as the sequencing depth. Both alpha- and beta-diversity were determined using the rarefied number of reads.

A permutational multivariate analysis of variance (PERMANOVA) was used to evaluate structural differences between rookeries with Monte Carlo tests and 4,999 permutations in PRIMER+P (v. 6; Clarke & Warwick, 2001) using Bray-Curtis, Jaccard, and weighted and unweighted UniFrac distances. However, the principal coordinate analysis (PCoA) results were visualized with EMPeror (Vázquez-Baeza et al., 2013) using a Jaccard distance of the most abundant ASV (>1% of the read frequency). Finally, to evaluate the principal bacterial taxa driving the dissimilarities, a similarity percentage (SIMPER) analysis of the ASVs detected within V6–V7 was performed in PRIMER+P (v. 6; Clarke & Warwick, 2001).

PICRUSt (v. 2.2.0) beta pipelines (Douglas et al., 2019) were used to predict the potential functional capabilities of the ASVs as well as their potential contributions to enriching the predicted ecological functions among all HRs. The abundances of the detected ASVs were normalized by the predicted *16S rRNA* gene copy number present in the closest reference genome. Subsequently, functional predictions were obtained with the predicted, orthologous genes in the Kyoto Encyclopedia of Genes and Genomes (KEGG) database of each ASV. For quality control purposes, the nearest sequenced taxon index (NSTI) was determined. This index reflects the accuracy of the functional predictions for each ASV by conveying the average genetic distance (measured as the number of substitutions per site) between an ASV and the *16S rRNA* of the most similar reference genome (Langille et al., 2013; de Voogd et al., 2015; Douglas et al., 2019). Following the suggested guidelines (Douglas et al., 2019), ASVs with NSTI values >2 were removed. To visualize the functional dispersion present among bacterial communities, PCoA plots were created with STAMP (v. 2.1.3; Parks et al., 2014) based on fourth-root normalized metabolic KEGG pathway counts. Significant functional differences between rookeries were evaluated by PERMANOVA with Monte Carlo tests based on 4,999 permutations in PRIMER+P (v. 6; Clarke & Warwick, 2001).

## Results

### Sequencing results

The yield of each HR was evaluated by read counts, and microbial composition and diversity were determined. A total of 281,630 raw reads (254-bp average length) were sequenced, with 266,177 (93.4%) reads meeting quality control test standards and being assigned to 1,852 ASVs. The number of detected ASVs and reads for each HR (read number reported in parentheses) were 107 (55,861), 87 (45,981), 53 (71,467), 114 (87,249), 7 (89,181), and 17 (33,398) for V2, V3, V4, V6–V7, V8, and V9, respectively. The rarefaction curves were asymptotic in shape, suggesting that the major fraction of the bacterial communities were sampled for V2, V3, V4, and V6–V7; however, the ASVs detected for V8 and V9 were mostly assigned to *Proteobacteria*, suggesting that the bacterial community was not entirely characterized with those regions (Fig. S1).

### Bacterial structural compositions among HRs

**Figure 2:**
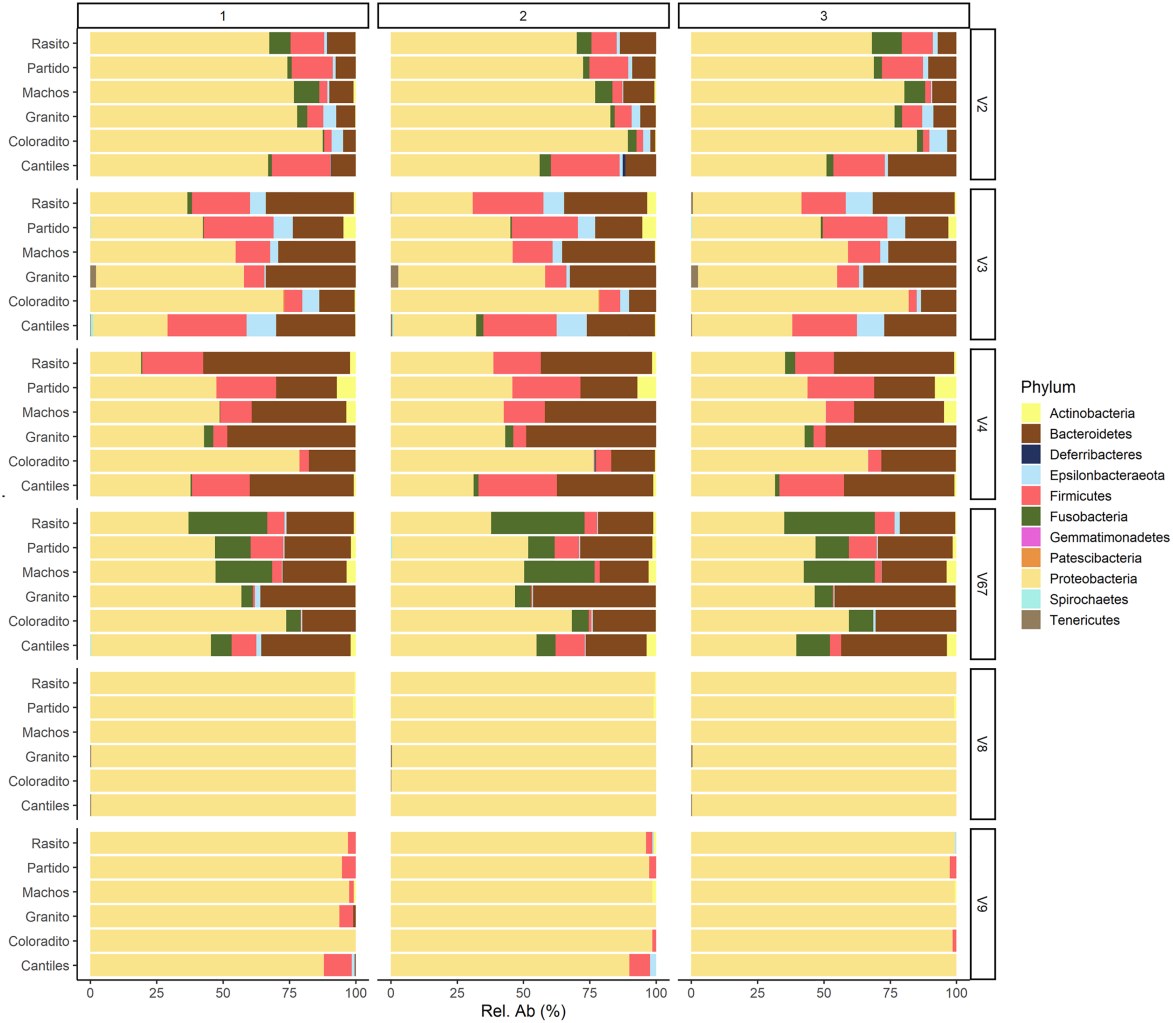
Bacterial abundances. Major bacterial taxa of the gut microbiota of sea lions from six breeding rookeries (Rasito, Partido, Machos, Granito, Coloradito, and Cantiles) in three DNA samples (1 to 3) detected from six hypervariable regions (HRs; V2–V9) of *16S rRNA*.

**Figure 3:**
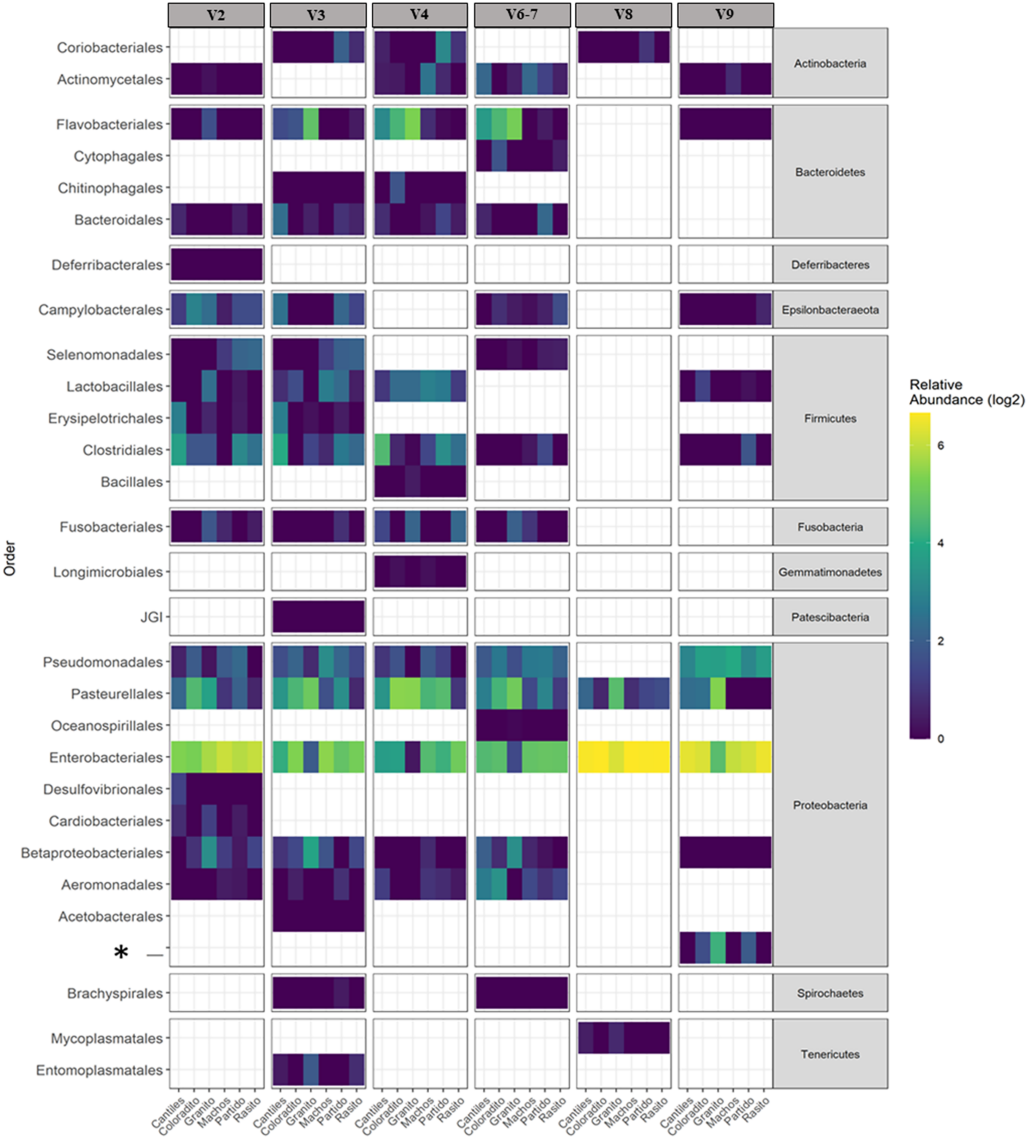
Bacterial distribution. Heat map based on the normalized abundances of predominant bacterial orders (cutoff > 1%) detected for each hypervariable region (HR; V2–V9) and among all sampling rookeries (Rasito, Partido, Machos, Granito, Coloradito, and Cantiles). An asterisk (*) refers to an unassigned taxonomic bacterial order of the Protobacteria phylum.

The capacity of each HR to determine the composition of the fecal microbiome at different taxonomic levels was analyzed. At the phylum level (average read frequency in parentheses) and among all HRs, Proteobacteria (up to 100%) predominated followed by Bacteroidetes (up to 30.26%) and Firmicutes (up to 14.73%). Nevertheless, distinctive arrangements were observed among HRs (Fig. 2). For example, Fusobacteria were mostly detected with V6–V7, while Deferribacteres and Gemmatimonadetes were exclusively detected with V2 and V4, respectively (Fig. S2). Similar results were also observed at lower taxonomic levels. Distinctive abundance rearrangements of the 29 predominant bacterial orders (>1% abundance cutoff; Fig. 3) were observed for each tested HR. Given the average values of all ecological indices considered in this study, the highest bacterial community diversity was generally detected with V6–V7, whereas the least complex bacterial community was detected with V8 (Table 1).

**Table 1:** Bacterial diversity among rookeries.

	Cantiles	Coloradito	Granito	Machos	Partido	Rasito	
V2	32.67	27.00	29.00	23.00	37.00	27.33	Av. ASV
	3.35	2.84	2.79	2.75	3.07	2.76	Av. Shannon
	7.89	6.71	7.10	6.57	7.65	7.02	Av. FD
V3	38.67	29.33	25.33	25.00	34.00	34.00	Av. ASV
	4.44	3.79	3.66	3.74	4.52	4.09	Av. Shannon
	5.66	4.41	3.64	4.62	5.80	4.96	Av. FD
V4	26.67	28.00	18.33	23.67	22.67	21.33	Av. ASV
	3.88	3.46	2.86	3.28	3.73	3.17	Av. Shannon
	4.16	3.94	3.32	3.72	3.84	3.89	Av. FD
V6–V7	42.33	37.67	35.67	27.67	39.00	40.33	Av. ASV
	4.67	4.24	4.20	4.08	4.51	4.20	Av. Shannon
	5.74	5.09	4.74	4.80	5.70	5.36	Av. FD
V8	4.67	4.33	5.00	4.00	5.33	4.67	Av. ASV
	1.68	1.29	1.51	1.55	1.55	1.54	Av. Shannon
	1.67	1.62	1.71	1.62	2.15	1.94	Av. FD
V9	8.67	9.33	8.33	6.67	8.67	6.67	Av. ASV
	1.89	1.78	2.42	1.98	1.77	1.10	Av. Shannon
	2.03	1.81	1.86	1.87	2.00	1.90	Av. FD

**Note:**

Gut microbiome alpha diversity among different sea lion colonies (Cantiles, Coloradito, Granito, Machos, Partido, and Rasito) determined by the average number of observed amplicon sequence variant (ASV; Av. ASV), Shannon diversity (Av. Shannon), and phylogenetic diversity (Av. FD) index values for each *16S rRNA* region (V2–V9).

### Bacterial compositions between rookeries

**Figure 4:**
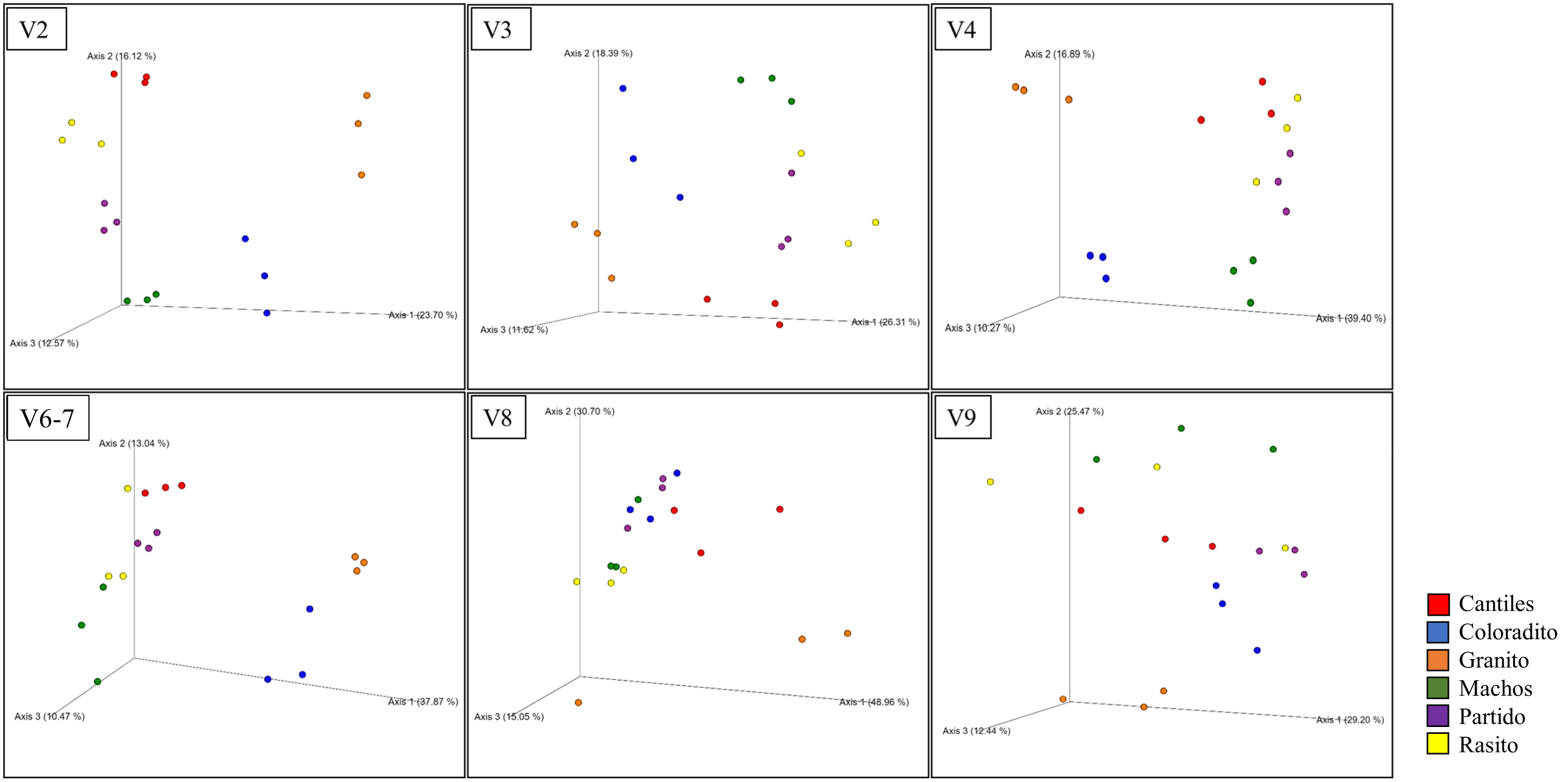
Bacterial structural variation. Principal coordinate analysis (PCoA) showing microbiome groupings among rookeries (Cantiles, Coloradito, Granito, Machos, Partido, and Rasito) for each analyzed hypervariable region (HR) of *16S rRNA* (V2–V9).

The predominant orders among all rookeries were Enterobacteriales and Pasteurellales (Fig. 3); however, structural differences were also observed between sampling areas. Specifically, the SIMPER analysis outcomes (Table S1 from A to O) suggested that the major fraction of geographic variability was explained by shifts in the relative abundance of common ASVs mainly assigned to Proteobacteria, Bacteroidetes, and Fusobacteria phyla rather than the presence of exclusive bacterial ASVs among rookeries. Significant differences in bacterial communities were observed between all distance matrices (PERMANOVA; pseudo-f: >2.33; *p* < 0.0158) for V3, V4, and V6–V7. Moreover, within the V2 region, no significant differences were observed using Bray-Curtis distances between Coloradito and other regions (PERMANOVA; pseudo-f: <0.19; *p* > 0.072) nor were significant differences observed with the V8 and V9 regions (PERMANOVA; pseudo-f: <1.69; *p* > 0.11). The results of the PCoA based on Jaccard distance are reported in Fig. 4.

Despite not observing unambiguous geographic patterns, higher bacterial α-diversity was generally detected in Cantiles and Partido compared to the values observed in Coloradito, Granito, and Machos (Table 1).

### Functional microbiome capabilities

Functional predictions were estimated by using 7 (V8) to 110 (V6–V7) ASVs. During these analyses, two, three, and four ASVs were removed from the V2, V4, and V6–V7 libraries, respectively, as these ASVs presented NSTI values >2. For the remaining *16S rRNA* regions, all detected ASVs presented NSTI values <2. A higher number of functional pathways was observed for the V3, V6–V7, and V4 regions for which 147, 142, and 139 pathways were predicted, respectively (Tables S2–S7). Overall, functional capabilities (gene count percentages in parentheses) were mainly assigned to the metabolic pathways (up to 73.44%) of carbohydrate metabolism (14.92%), cofactor and vitamin metabolism (12.44%), and amino acid metabolism (13.02%). The major bacterial families responsible for assigning these functions were Enterobacteriaceae (up to 94.67%) and Pasteurellaceae (up to 18.7%). Additional bacterial families with relatively high functional contributions (threshold > 3%) in at least one HR are reported in Table 2.

**Table 2:** Bacterial functional contributions.

		V2		V3		V4		V6-7		V8		V9	
Phylum	Family	abb.%	Met.con.%	abb.%	Met.con.%	abb.%	Met.con.%	abb.%	Met.con.%	abb.%	Met.con.%	abb.%	Met.con.%
*Bacteroides*	*Bacteroidaceae*	15.8	6.68	18.59	12.98	23.23	18.58	11.88	10.94	nd	nd	nd	nd
	*Porphyromonadaceae*	nd	nd	6.83	4.68	6.98	5	5.91	4.79	nd	nd	nd	nd
	*Rikenellaceae*	<3	<3	4.46	5.06	<3	<3	<3	<3	nd	nd	nd	nd
	*Weeksellaceae*	nd	nd	<3	<3	11.69	12.69	6.77	9.74	nd	nd	<3	<3
*Epsilonbacteraeota*	*Campylobacteraceae*	4.45	<3	4.56	3.04	nd	nd	<3	<3	nd	nd	<3	<3
*Firmicutes*	*Lachnospiraceae*	<3	<3	11.01	8.44	4.09	3.01	3.63	<3	nd	nd	nd	nd
	*Ruminococcaceae*	8.9	3.6	5.93	6.22	5.95	6.24	<3	<3	nd	nd	<3	<3
	*Streptococcaceae*	<3	<3	nd	nd	nd	nd	nd	nd	nd	nd	3.94	<3
*Fusobacteria*	*Fusobacteriaceae*	8.07	3.39	<3	<3	nd	nd	16.22	13.27	nd	nd	nd	nd
*Protobacteria*	*Neisseriaceae*	3.94	<3	<3	<3	nd	nd	<3	<3	nd	nd	<3	<3
	*Enterobacteriaceae*	23.74	64.1	9.07	27.14	9.92	16.6	22.01	28.32	89.18	94.67	34.61	71.02
	*Pasteurellaceae*	11.84	5.43	10.57	7.89	23.25	18.7	14.8	13.35	10.63	5.28	7.49	7.14
	*Moraxellaceae*	5.91	5.91	<3	<3	6.97	6.29	<3	<3	nd	nd	16.34	6.95
	*Pseudomonadaceae*	<3	<3	<3	<3	<3	<3	5.5	5.71	nd	nd	26.65	9.5

**Note:**

Abundances (abb.%) and contributions to metabolic pathways (Met.con.%) of each of the main bacterial phyla and families (cutoff > 3%).

**Figure 5:**
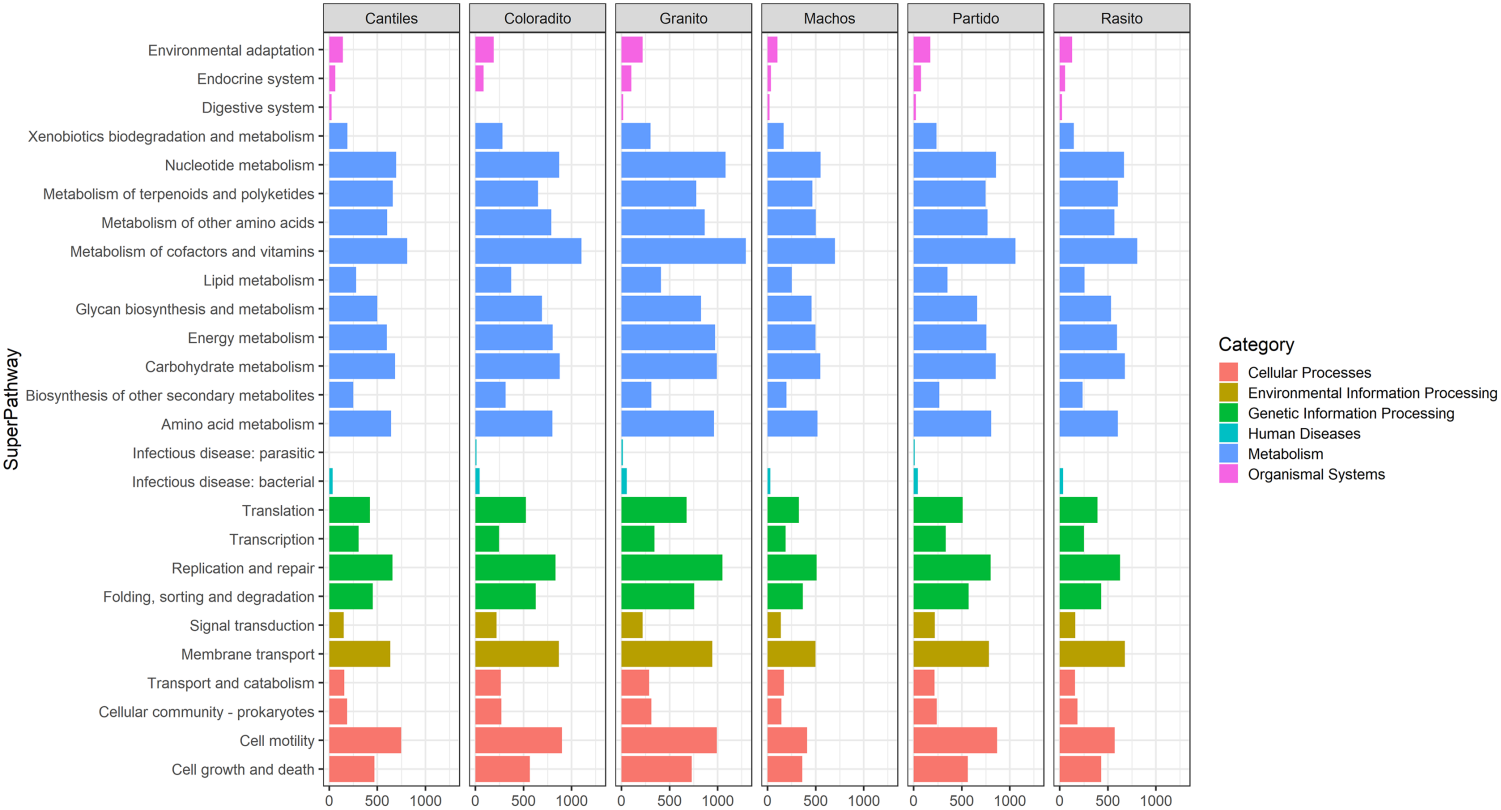
Bacterial functional profiles. Predicted functional profiles among hypervariable regions (HRs: V2–V9) according to Kyoto Encyclopedia of Genes and Genomes (KEEG) level 1 (Category) and level 2 (SuperPathway).

**Figure 6:**
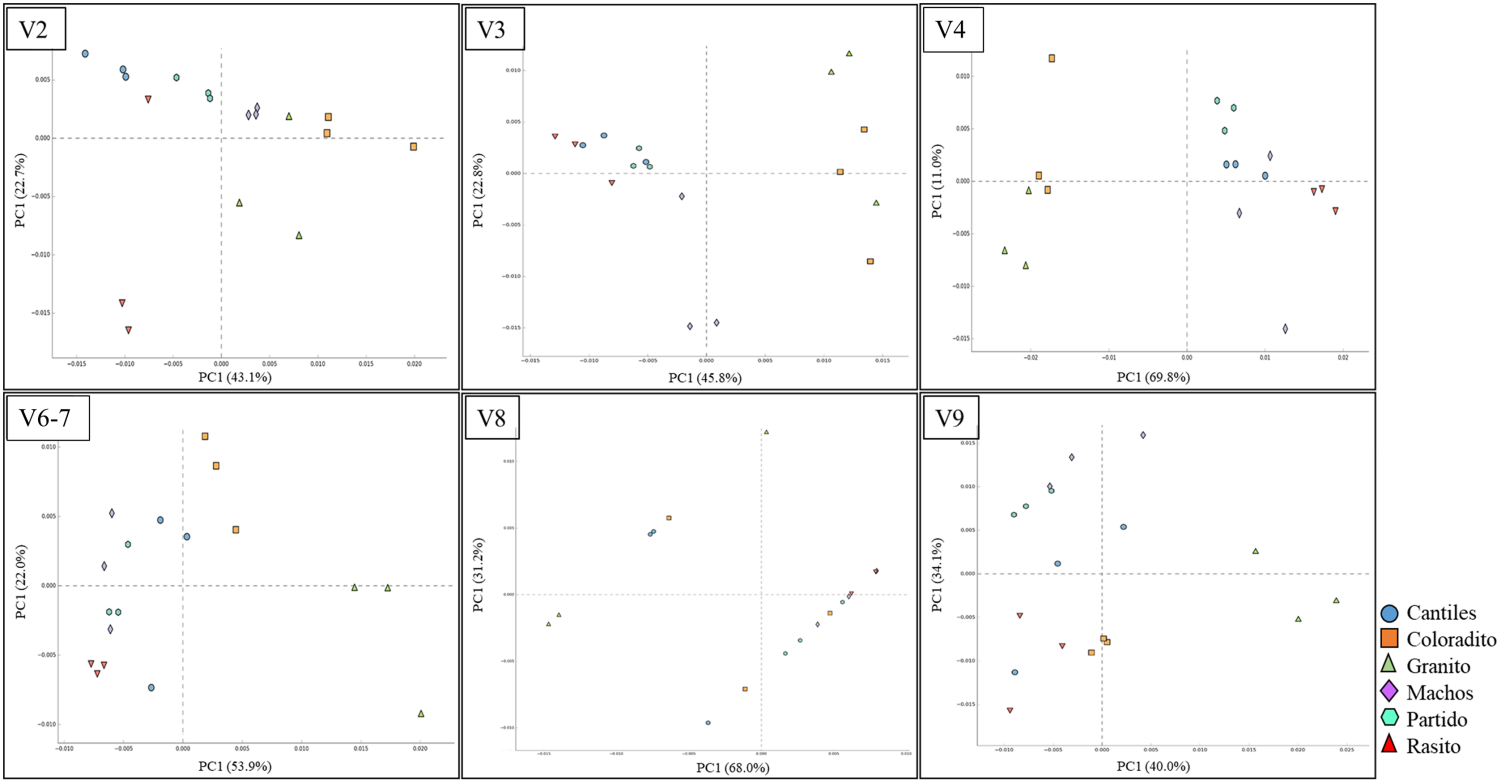
Bacterial functional variation. Principal coordinate analysis (PCoA) based on the normalized pathway abundances for each hypervariable region (HR) of *16S rRNA* (V2–V9).

Similar abundances in functional pathway profiles were observed among rookeries (KEGG hierarchical level 1; Fig. 5). Notably, seven bacterial genera were associated with the assigned metabolic activities and included *Escherichia-Shigella* (~22%), *Fusobacterium* (~16.22%), *Otariodibacter* (~14%), *Bacteroides* (11.88%), *Ornithobacterium* (6.77%), *Porphyromonas* (5.91%), and *Pseudomonas* (5.5%). Moreover, the metabolic functions in Coloradito and Granito were mostly enriched by *Otariodibacter* and *Ornithobacterium*, whereas *Escherichia-Shigella* and *Bacteroides* mainly contributed to the same functions in Macho, Partido, Rasito, and Cantiles, as did *Fusobacterium* in Macho and Rasito. The contributions of *Pseudomonas* and *Porphyromonas* were consistent among all rookeries. The PCoA results based on the normalized count numbers of the pathways at KEGG hierarchical level 3 suggested functional splits between most of the sampled rookeries for the V2, V3, V4, and V6–V7 regions although not for V8 and V9 (Fig. 6). Nevertheless, the PERMANOVA results revealed consistent differences only between Granito and Partido and Granito and Rasido for the V4 region (pseudo-f: >2.26; *p* < 0.0482).

## Discussion

### Bacterial taxa abundance in pinnipeds

Technological advances in high throughput sequencing have provided unprecedented opportunities to characterize bacterial communities from almost any type of environmental DNA sample, including samples from the GI tracts of host species. However, accurately characterizing these communities can be challenging due to the unequal amplification of bacterial taxa during PCR (Brooks et al., 2015). Moreover, the main limitation of metabarcoding surveys can be the incompleteness of reference databases (Martinez-Porchas et al., 2017; Gold et al., 2021). The latter is particularly evident for external HRs, which are known to have incomplete reference sequences (Martinez-Porchas et al., 2017). Moreover, metabarcoding surveys measure relative rather than absolute biological abundance (Barlow, Bogatyrev & Ismagilov, 2020). To circumvent or at least minimize these difficulties in this study, we integrated the information from six HRs of *16S rRNA* to obtain high-resolution microbial community profiles.

The combined information from the tested HRs suggests that the bacterial communities in the rectal samples of California sea lion pups are mainly composed of five phyla: Proteobacteria, Bacteroidetes, Epsilonbacteraeota, Firmicutes, and Fusobacteria. Despite most of these taxa being common and important for pinniped health, variations in their abundance remains a topic in need of discussion. For example, the findings from this study suggest that Firmicutes may cumulatively constitute up to 30% of GI bacterial communities in sea lions; however, Delport et al. (2016) reported that Firmicutes may constitute approximately 76% of the GI bacterial communities in Australian sea lions (*Neophoca cinerea*). Comparisons between studies should be made cautiously due to differences in methodology (*e.g*., variations in DNA extraction methods, PCR-targeted *16S rRNA* regions, and downstream bioinformatic methods) and other factors (*e.g*., environment, health, inheritance, or age). Nevertheless, several studies that have used the universal primers 27F (5′-AGAGTTTGATCCTGGCTCAG-3′; *e.g*., Ludwig, Mittenhuber & Friedrich, 1993) and 519R (5′-GWATTACCGCGGCKGCTG-3′; *e.g*., Ruff-Roberts, Kuenen & Ward, 1994) that span the V1–V3 regions have usually identify Firmicutes as the predominant bacterial species in both pinniped (Nelson et al., 2013; Acquarone, Salgado-Flores & Sundset, 2020) and non-pinniped host species (Cicala et al., 2018; Brown, Wiens & Salinas, 2019). Conversely, when alternative primer sets targeting V4 have been implemented, the reported Firmicutes abundances are more consistent with the findings of this study, as evidenced by *Phoca vitulina richardii* (~26%), *Leptonychotes weddellii* (~23%), and *Arctocephalus pusillus* (~25%) abundance (Banks, Cary & Hogg, 2014; Pacheco-Sandoval et al., 2019; Bai et al., 2021).

Discrepancies in bacterial taxa abundance can be extended to include Proteobacteria and Epsilonbacteraeota. To the best of our knowledge, Epsilonbacteraeota is one of the predominant bacterial taxa in the GI microbiota of Cape fur seals within V4 (Bai et al., 2021); however, this bacterial group has not been previously identified in the GI bacterial community of *Zalophus californianus* nor those of other sea lion species. Moreover, in the present study, Epsilonbacteraeota was largely detected within the V3 region. Together, these findings suggest that Epsilonbacteraeota may be considered a predominant bacterial group in sea lions, even though we speculate that this bacterial taxon has not been previously detected in sea lions given that V3 remains a poorly utilized region in metabarcoding surveys. Nevertheless, more evidence is needed to support or disprove this hypothesis. Our results also identified Proteobacteria as one of the main taxa in bacterial communities, with abundances as high as 60% in the fecal samples of sea lion pups. Conversely, several authors have reported abundances of this phylum as low as 1–2% based on the genetic information from V1–V2 or V3–V5 (Smith et al., 2013; Bik et al., 2016). Thus, further research is required to determine the exact proportion of Proteobacteria in pinniped GI tracts.

Studies examining GI bacterial diversity using six HRs in sea lions or other pinniped species are lacking; thus, our results may encourage a reevaluation of previously reported bacterial abundance profiles in pinnipeds. Moreover, the proposed observations may provide starting points for further investigations, as the most comprehensive description of the bacterial community was obtained by integrating information from the V3 and V6–V7 regions. These HRs may reflect GI changes better than the other *16S rRNA* regions (Vargas-Albores et al., 2017); hence, future bacterial surveys of sea lions, and possibly those of other pinniped species, may benefit from sequencing approaches employing the V3 and V6–V7 regions.

### Bacterial community variation between rookeries

Given that distinctive HR-related bacterial consortia were detected, we evaluated which among the tested HRs might be most suitable for identifying structurally related microbiome features, such as geographic changes or functional capabilities (reported in the next paragraph). Even after considering the low number of samples processed for this analysis (*n* = 3 per rockery), consistent structural variation among rookeries was observed among V3, V4, and V6–V7, whereas marginal geographic changes were observed within V8 and V9.

Notably, structural geographic differences were mainly related with variations in the relative abundances of Proteobacteria, Fusobacteria, and Bacteroidetes rather than through the acquisition of new bacterial species. This observation may further support the existence of a bacterial core in pinnipeds (Glad et al., 2010; Nelson et al., 2013; Numberger et al., 2016).

Geographic GI microbiota patterns have been reported for sea lions as well as other pinnipeds, and these are thought to be associated with several factors, including diet, site fidelity, and colony density (Nelson et al., 2013; Delport et al., 2016; Pacheco-Sandoval et al., 2019). Although we did not directly address the contributions of these factors on GI bacterial diversity, our experimental design may be useful for explaining additional causes of local variations in GI microbiota. Indeed, we analyzed GI samples from pups 2–3 months old. At this age, sea lion pups are not able to move between colonies and are still dependent on maternal feeding. Therefore, the observed GI geographic variation in pups may reflect the local preferences of their mothers. If accepted, this finding may also suggest that GI microbiomes in pups are vertically inherited from mother to offspring at birth and as a result of nursing, as has recently been proposed for humans (Blekhman et al., 2015; Duranti et al., 2017; Meehan et al., 2018) and other mammalian species (Antwis et al., 2018), although additional research is needed to support this hypothesis. Further, our findings support the hypothesis that once an initial microbiota core is established, it may persist to adulthood (Nelson et al., 2013), even though external factors may modulate the abundance of certain bacteria that promote the most beneficial configuration for the host. For example, Smith et al. (2013) reported that milk-based diets in seal pups promote the growth of Lactobacilli, which were present in lower abundances in adults with solid, marine-based diets.

Finally, our results do not support the observation that higher microbial diversity is usually detected in high-density colonies (Nelson et al., 2013; Delport et al., 2016). Relatively high values of bacterial community diversity were observed in Partido, one of the colonies with the lowest number of individuals (Szteren, Aurioles & Gerber, 2006; Masper et al., 2019). This observation suggests that colony density *per se* is not a force that shapes GI bacterial communities and that individuals with close ecological ties (*e.g*., similar diets) are more likely to converge and exhibit similar microbiome compositions, regardless of colony density.

### Microbiome functional predictions

Given that we detected distinctive HR-related bacterial consortia, we evaluated if these alternative arrangements could lead to different functional capabilities of the microbiota. Despite the different number of molecular pathways identified among HRs, no significant HR-driven changes were apparent. In addition, similar functional profiles were also observed between rookeries. The simplest explanation of such consistent results may lie in the proposed methodological approach. The results of comparative analyses have recently suggested that the sensibility of PICRUSt and other bioinformatics tools for *in silico* functional predictions of microbial community is largely limited in non-human samples (Sun, Jones & Fodor, 2020). Nevertheless, the high degree of genetic similarity between the detected ASVs and reference genomes (highlighted by the low NSTI values) may indicate reliable prediction accuracy, and thus additional explanations may be considered.

In this context, we observed that the majority of the predicted functional activities were enriched by seven bacterial genera: *Escherichia*, *Fusobacterium*, *Otariodibacter*, *Bacteroides*, *Ornithobacterium*, *Porphyromonas*, and *Pseudomonas*. Hence the GI microbiota of sea lion pups may be characterized by a high degree of functional redundancy (*i.e*., different bacterial species bearing similar ecological functions). As has been reported in other species, functional redundancy appears to be an intrinsic property of mammalian GI ecosystems that sustains homeostatic conditions, which ultimately guarantees host health (Lavery et al., 2012; Pérez-Cobas et al., 2013; Moya & Ferrer, 2016). This condition may be particularly evident in sea lion pups given that their GI microbiomes are primarily involved in metabolic activities. According to recent genomic annotations, the genomes of Bacteroidetes and Fusobacteria are known to present a high number of genes involved in the degradation of glycans, proteins, and complex oligosaccharides (Wang et al., 2016; Birhanu et al., 2019). Accordingly, these genera may confer ecological advantages in sea lions by increasing fat deposition, which is necessary for thermal isolation in the aquatic environment, where heat transfer is several times faster than the terrestrial environments and supplies energy (Lavery et al., 2012; Acquarone, Salgado-Flores & Sundset, 2020).

Notably, some of these genera are known to be pathogenic members of human GI communities; however, their high abundance in predicted KEGG pathways suggests mutualistic relationships with sea lion pups. We speculate that sea lions and other pinnipeds species may act as natural reservoirs for these human pathogenic bacteria (Stewart et al., 2008; Zheludkov & Tsirelson, 2010; Sato et al., 2019), which switch from being commensals to pathogen species after their introduction into “non-native” host species, although additional research is needed to support this hypothesis.

## Conclusions

As hypothesized, distinctive structural bacterial arrangements were observed for each of the analyzed HRs of *16S rRNA*. Consequently, bacterial community characterizations may be more accurately achieved by a multi-locus approach. The use of a single HR of *16S rRNA* may lead to misleading structural results that hamper the identification of the intrinsic characteristics of the microbiota. Given that studies of GI bacterial diversity that target six HRs in sea lions or other pinniped species are lacking, our findings may encourage a reevaluation of previously reported bacterial abundance profiles in pinnipeds. In this context, this study may also provide a starting point for further investigations, as the most comprehensive bacterial community information was retrieved using the synergistic information of the V3 and V6–V7 regions.

## Supplemental Information

10.7717/peerj.13235/supp-1 Supplemental Information 1 Bacterial diversity and sample size relationship. Rarefaction curves based on the cumulative number of observed amplicon sequence variants (ASVs) for each hypervariable region (HR; V2–V9).

10.7717/peerj.13235/supp-2 Supplemental Information 2 Specific abundances among hypervariable regions. Relative read number (N reads) for each of the main bacterial taxa (percentage cutoff of 3%) detected with each hypervariable region (HR; V2–V9) of the *16S rRNA* gene.

10.7717/peerj.13235/supp-3 Supplemental Information 3 Abundance differences of amplicon sequence variants. Abundance differences of amplicon sequence variants (ASVs) detected with V6–V7 region among sampling rookeris. Column abbreviations refer to average abundance (Av. Abund), average dissimilarity (Av. Diss), dissimilarity percentage of the contribution of each bacterial species (Contib%), and cumulative dissimilar contribution (Cum%).

## Additional Information and Declarations

### Data Availability

The following information was supplied regarding data availability: The sequences are available at BioProject: PRJNA715917.
