# Supplementary figures and images for: Multi-locus evaluation of gastrointestinal bacterial communities from Zalophus californianus pups in the Gulf of California, México
Source: PeerJ. 2022 Jul 8;10:e13235. doi: 10.7717/peerj.13235 (PMC9272818; doi:10.7717/peerj.13235)

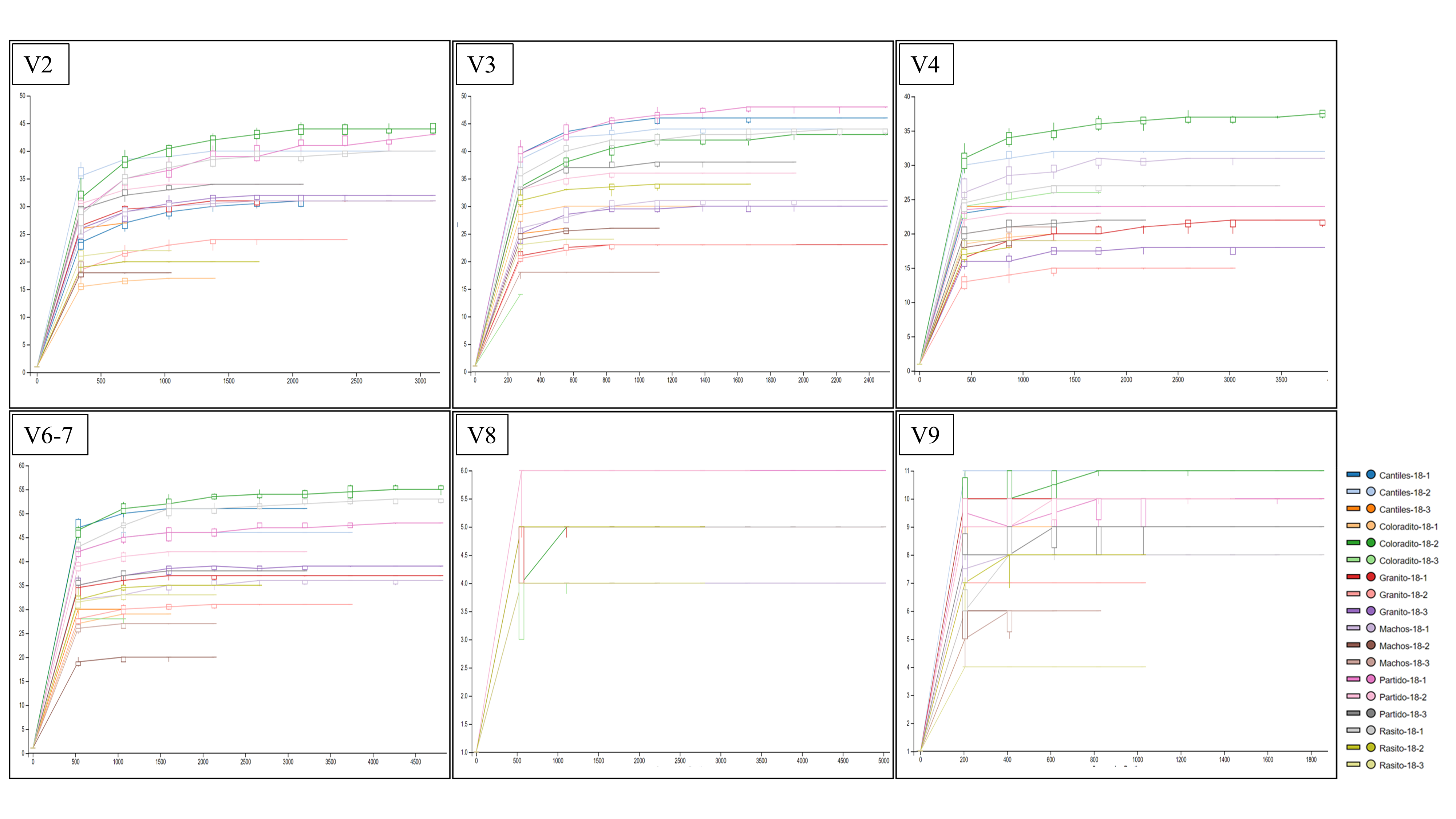

Supplement: Supplemental Information 1 — Rarefaction curves based on the cumulative number of observed amplicon sequence variants (ASVs) for each hypervariable region (HR; V2–V9). [file peerj-10-13235-s001.png]

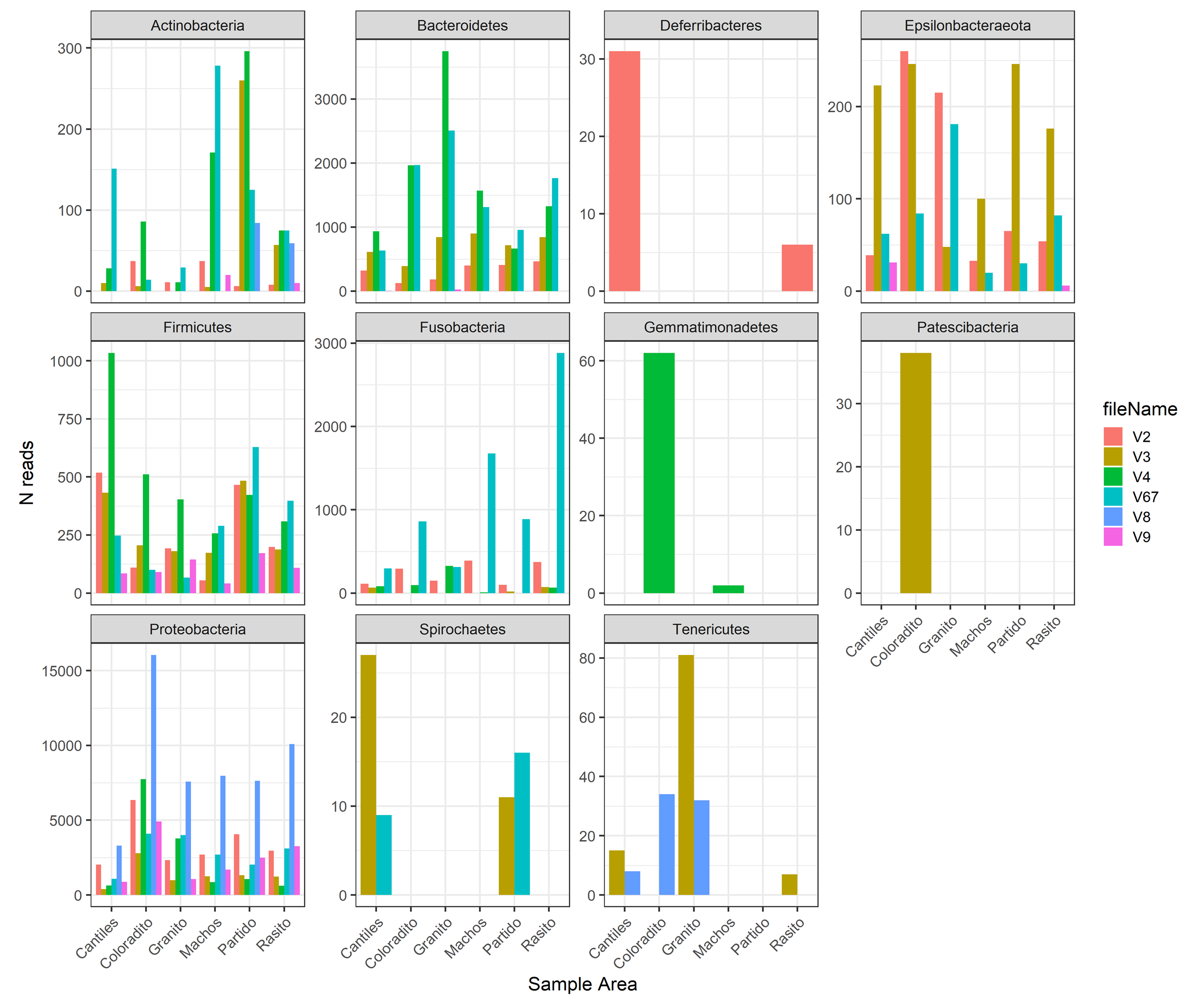

Supplement: Supplemental Information 2 — Relative read number (N reads) for each of the main bacterial taxa (percentage cutoff of 3%) detected with each hypervariable region (HR; V2–V9) of the 16S rRNA gene. [file peerj-10-13235-s002.png]
